# Prognostic Significance of PET/CT in Patients with Chronic Lymphocytic Leukemia (CLL) Treated with Frontline Chemoimmunotherapy

**DOI:** 10.3390/cancers12071773

**Published:** 2020-07-03

**Authors:** Marika Porrazzo, Emanuele Nicolai, Mara Riminucci, Candida Vitale, Marta Coscia, Lorenzo De Paoli, Angela Rago, Giulia Buscicchio, Giacomo Maestrini, Silvio Ligia, Alessio Di Prima, Alessandro Corsi, Roberto Caronna, Gianluca Gaidano, Francesca Romana Mauro

**Affiliations:** 1 Hematology, Department of Translational and Precision Medicine, Sapienza University, Via Benevento 6, 00161 Rome, Italy; marika.porrazzo@uniroma1.it (M.P.); maestrini@bce.uniroma1.it (G.M.); ligia@bce.uniroma1.it (S.L.); diprima@bce.uniroma1.it (A.D.P.); 2 Institute of Diagnostic and Nuclear Research, IRCCS SDN, 80143 Naples, Italy; enicolai@sdn-napoli.it; 3 Department of Molecular Medicine, “Sapienza” University of Rome, 00161 Roma, Italy; mara.riminucci@uniroma1.it (M.R.); alessandro.corsi@uniroma1.it (A.C.); 4 Division of Hematology, A.O.U. Città della Salute e della Scienza di Torino and Department of Molecular Biotechnology and Health Sciences, University of Turin, 10124 Torino, Italy; candida.vitale@unito.it (C.V.); marta.coscia@unito.it (M.C.); 5 Division of Hematology, Department of Translational Medicine, University of Eastern Piedmont, 28100 Novara, Italy; lorenzo.depaoli@med.unipmn.it (L.D.P.); gaidano@med.unipmn.it (G.G.); 6 UOSD Hematology, ASL Roma 1, 00193 Rome, Italy; rago@bce.uniroma1.it; 7 Department of Psychology, Catholic University of the Sacred-Heart, 20123 Milan, Italy; giulia.buscicchio@unicatt.it; 8 Department of Surgical Sciences, Sapienza University, 00161 Rome, Italy; roberto.caronna@uniroma1.it

**Keywords:** chronic lymphocytic leukemia, PET/CT, survival

## Abstract

The role of positron emission tomography/computed tomography (PET/CT) in identifying Richter Syndrome (RS) is well established, while its impact on the survival of patients with chronic lymphocytic leukemia (CLL) has been less explored. The clinical characteristics and PET/CT data of 40 patients with a biopsy-proven CLL who required frontline chemoimmunotherapy, FCR (fludarabine, cyclophosphamide, rituximab) in 20 patients, BR (bendamustine, rituximab) in 20, were retrospectively analyzed. Standardized uptake volume (SUV_max_) values ≥ 5 were observed more frequently in patients with deletion 11q (*p* = 0.006) and biopsies characterized by a rate of Ki67 positive cells ≥ 30% (*p* = 0.02). In the multivariate analysis, the presence of large and confluent PCs emerged as the only factor with a negative impact on progression-free survival (PFS), and overall survival (OS). Deletion 11q also revealed a significant and independent effect on PFS. SUV_max_ values ≥ 5 showed no statistical impact on PFS while in multivariate analysis, they revealed a significant adverse impact on OS (median survival probability not reached vs. 56 months; *p* = 0.002). Moreover, patients with higher SUV_max_ values more frequently developed Richter Syndrome (*p* = 0.015). Our results show that higher SUV_max_ values identify CLL patients with a pronounced rate of proliferating cells in the lymph-node compartment, inferior survival, and an increased risk of developing RS.

## 1. Introduction

Chronic lymphocytic leukemia (CLL) is the most common type of leukemia in adults in the Western World. Patients with CLL show heterogeneous outcomes ranging from an indolent to an aggressive clinical course [1]. Approximately 2–10% of CLL patients develop additional genetic lesions leading to an increased risk of Richter Syndrome (RS), an aggressive lymphoma characterized by a high proliferative pattern [2,3,4]. Several studies have demonstrated the important role of positron emission tomography/computed tomography (PET/CT) in detecting RS [5,6,7,8,9]. This technique can distinguish tissues with a low 1*8F*-*fluorodeoxyglucose* (*18F*-*FDG*) uptake, such as CLL, from those with a higher metabolic pathway, such as aggressive lymphomas and second malignancies [10,11,12,13,14,15]. Higher standardized uptake volume (SUV_max_) values ≥5 or ≥10, revealed a high sensitivity and specificity in detecting RS in patients treated with chemoimmunotherapy [5,6,7,8,9]. On this basis, PET/CT has been considered a useful tool to identify both the presence of high metabolic disease and the optimal site for a diagnostic biopsy [5,6,7,8,9,16]. However, PET cannot replace histology in the diagnostic assessment of tissues with a high 18F-FDG uptake.

In different studies, high SUV_max_ values have also been associated with poorer outcomes [6,7,17,18]. In a retrospective study by Falchi et al. that included 764 CLL patients who underwent PET/CT in the susceptibility of a more aggressive disease or as clinical staging before different treatment lines, 332 (43%) patients underwent the biopsy of the involved lymph node or other tissue [6]. RS was diagnosed in 95 (28.6%) patients while CLL was confirmed histologically in 237 (71.4%). In this study, the SUV_max_ values ≥ 10 identified patients with RS and were associated with inferior survival. Moreover, the patients with confirmed CLL who showed histologic features of a more aggressive disease, showed higher median SUV_max_ values, 6.8, than those of the patients with a histologic indolent disease, 3.7. Similarly, in the study by Michallet et al. SUV_max_ values ≥ 10 identified patients with RS and were associated with significantly lower survival. In this study, patients with aggressive CLL showed higher median SUV_max_ values, 4.5, compared to those with a stable disease, 2.5 [9]. A prior study of our group included 90 patients in different treatment lines, who underwent PET/CT followed by a biopsy of the involved lymph nodes or other tissues in the suspicion of RS or a secondary malignancy [8]. Median SUV_max_ values were 14.6 in patients with diffuse large B-cell lymphoma, 7 in those with Hodgkin lymphoma, and 3.5 in patients with the diagnosis of CLL/small lymphocytic lymphoma. In this study, ten patients with previously untreated CLL and SUV_max_ cutoff ≥5 showed a significant inferior survival probability than those with lower SUV_max_ values. This finding might suggest an unfavorable outcome of CLL patients with a more marked proliferative behavior measured by PET/CT.

While the role of PET/CT in identifying the presence of RS is well established, its prognostic role in defining the outcome of patients with a biopsy-proven CLL has been less explored. In particular, the clinical significance of PET/CT as a prognosticator has not been sufficiently investigated in patients requiring front-line chemoimmunotherapy.

To define whether PET/CT may have prognostic significance in CLL, we retrospectively analyzed the clinical characteristics and outcome of 40 patients with a biopsy-proven CLL who required front-line chemoimmunotherapy.

## 2. Results

### 2.1. Patient Characteristics

The clinical and biological characteristics of patients are described in Table 1. The median time from PET/CT scan to biopsy was 26 days (range 14–42) and the median follow-up of patients from biopsy, 66 months (2–106). The median age at biopsy was 62 years (range 35–92). Unmutated immunoglobulin heavy chain variable region genes IGHVstatus was present in 54% of patients, and deletion 11q in 19%. The presence of a *TP*53 disruption was observed in 7/37 tested patients (19%). The fluorescent in situ hybridization FISH analysis revealed deletion 17p in 3/31 analyzed patients while *TP*53 mutation was detected in 6/37 tested patients (two patients with deletion 17p also had *TP*53 mutation). FCR (fludarabine, cyclophosphamide, rituximab) was given in 20 patients, and BR (bendamustine, rituximab) in 20.

### 2.2. PET/CT and Biopsy

The median SUV_max_ value recorded by PET was 3.5 (range, 2.1–9), with values ≥ 5 in 13 (33%) patients and <5 in 27 (67%). The biopsy site fully matched with that of the highest SUV_max_ value in 32 (80%) patients. In eight (20%) patients with the highest uptake projected on abdominal nodes, the biopsy targeted the largest lymph node easier to access.

### 2.3. Distribution of Clinical and Biologic Characteristics According to the SUV_max_ Value

In univariate analysis, SUV_max_ values ≥ 5 were observed more frequently in patients with biopsies characterized by a rate of Ki67 positive cells ≥ 30% (*p* = 0.02) and in those with deletion 11q (*p* = 0.006), while no significant differences in the SUV_max_ values were detected for other genetic lesions (Table 1).

### 2.4. Outcomes of Patients According to the SUV_max_ Values

Response to frontline therapy was observed in most patients [overall response rate (ORR), 36/40, 90%]. No significant differences in the overall response (OR) and complete response (CR) rates were associated with the SUV_max_ values recorded at baseline (SUV_max_ values < 5 vs. ≥5: ORR, 93% vs. 85%; *p* = 0.43; CR, 44% vs. 46%; *p* = 0.93; Table S1). After a median time of 34 months (range, 10–74 months) from the biopsy, 17 (49%) patients developed CLL progression. No statistical differences according to the SUV_max_ levels were observed in the rate of patients who developed CLL progression (SUV_max_ value ≥ 5 or <5: CLL progression, 33% vs. 54%; *p* = 0.28; Table S1) or not.

Five (13%) patients developed RS after a median time of 15 months (range, 12–32 months) from the biopsy. A significantly higher proportion of patients with SUV_max_ values ≥ 5 than those with SUV_max_ < 5 developed RS (SUV_max_ values ≥ 5 vs. <5; Richter Syndrome, 31% vs. 4%; *p* = 0.015; Table S1). The majority of patients who developed RS showed at baseline histologic features suggesting a high proportion of proliferating cells in lymph nodes (Ki67 ≥ 30%,4/5 patients; large and confluent proliferation centers (PCs), 3/5 patients).

### 2.5. Survival

**Figure 1:**
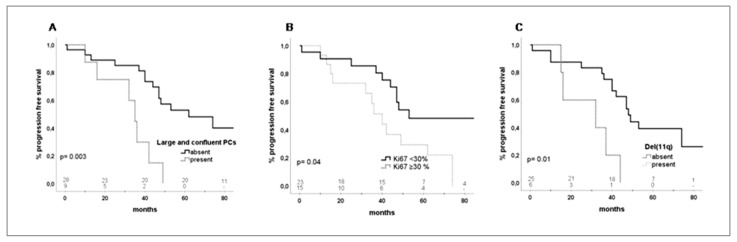
Progression-free survival (PFS) of patients with biopsy-proven CLL. (**A**) Thirty-six-month PFS according to the characteristics of proliferation centers (PCs): patients without large and confluent PCs, 85% (95% CI: 71.4–98.6) vs. with large and confluent PCs, 30% (95% CI: 12–78); *p* = 0.003. (**B**) Thirty-six-month PFS according to Ki67% expression: patients with Ki67% < 30, 85% (95% CI: 70–100) vs. patients with Ki67% ≥ 30, 51% (95% CI: 25.3–77.3); *p* = 0.04. (**C**) Thirty-six-month PFS according to deletion (11q): patients without del(11q), 75% (95% CI: 77.6–92.4) vs. patients with del(11q), 40% (95% CI: −1–81); *p* = 0.01.

The median and 3-year progression-free survival (PFS) were 48 months and 75%, respectively. In the univariate analysis, the SUV_max_ values showed no impact on PFS (3-year PFS, SUV_max_ < 5 vs. ≥ 5, 79 vs. 55%, *p* = 0.48; Table 2). The factors with a significant impact on PFS were deletion 11q (*p* = 0.01; hazard ratio (HR) 4.21 (95% CI: 1.3–13)), biopsies with the presence of large and confluent PCs (*p* = 0.003; HR 4.5 (95% CI: 1.6–12.1)) and ≥30% of Ki67 positive cells (*p* = 0.04; HR 2.41 (95% CI: 1–5.6)). A trend for statistical significance was observed for the IGHV mutational status (*p* = 0.07), while the presence of *TP*53 aberrations showed no significant impact on PFS (*p* = 0.7). However, in multivariate analysis, the presence of deletion 11q (HR 0.28 (95% CI: 0.08–0.99)) and large and confluent PCs in the lymph-node biopsies (HR 0.17 (95% CI: 0.03–0.94)) were the only factors that maintained significance (Figure 1; Table 2; Table S2).

**Figure 2:**
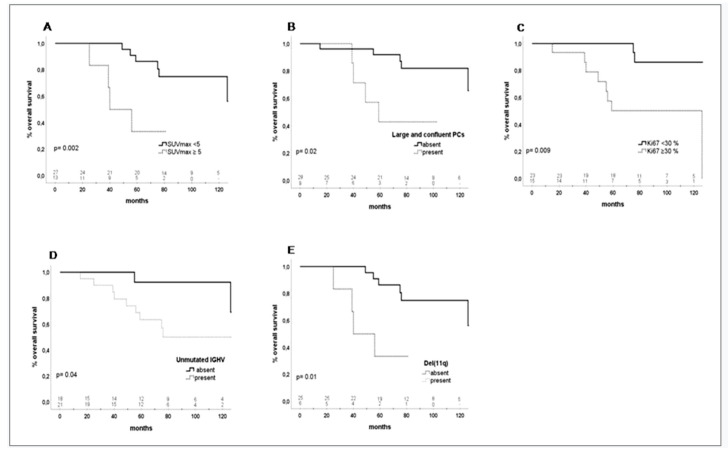
Overall survival (OS) of patients with biopsy-proven CLL. (**A**) Thirty-six-month OS according to the SUV_max_ < 5, 100% vs. SUV_max_ ≥ 5, 83% (95% CI: 64–102); *p* = 0.002. (**B**) Thirty-six-month OS according to the characteristics of proliferation centers (PCs): patients without large and confluent PCs, 96% (95% CI: 88.5–103.5) vs. with large and confluent PCs, 85% (95% CI: 89.3–110); *p* = 0.02. (**C**) Thirty-six-month OS according to Ki67% expression: Ki67% < 30, 100% vs. Ki67% ≥ 30, 93% (95% CI: 80.4–105.6); *p* = 0.009. (**D**) Thirty-six-month OS according to IGHV mutational status: IGHV mutated, 100% vs. IGHV unmutated, 90% (95% CI: 76.7–103.2), *p* = 0.04 (**E**) Thirty-six-month OS according to deletion (11q): patients without del(11q), 100% vs. patients with del(11q), 83% (95% CI: 53–113); *p* = 0.01.

Eleven (27%) patients died, 3/27 (11%) with SUV_max_ values < 5 and 8/13 (61%) with SUV_max_ values ≥ 5. The causes of death were disease progression in three cases, RS in four, cancer in two, cardiovascular in one, and a traumatic event in one. Median survival probability was not reached in patients with SUV_max_ values < 5 while it was significantly lower, 56 months, in those with SUV_max_ value ≥ 5 (*p* = 0.002; HR 0.12 (95% CI:0.3–0.5)) (Figure 2, Table 2).

A significantly inferior survival was also observed in the IGHV unmutated patients (*p* = 0.04; HR 0.20 (95% CI: 0.04–0.9)), in those with deletion 11q (*p* = 0.01; HR 5.49 (95% CI: 1.4–20.8)), with biopsies showing ≥ 30% of Ki67 positive cells (*p* = 0.009; HR 8.1 (95% CI: 1.7–38.6)), large and confluent PCs (*p* = 0.02; HR 5.17 (95% CI: 1.2–21.1)) (Figure 2, Table 2).

However, in the multivariate analysis, two factors maintained a significant adverse impact on OS, SUV_max_ values ≥ 5 (HR 6.48 (95% CI: 1.42–29.58)) and the presence of large and confluent PCs in the lymph nodes biopsies (HR 0.14 (95% CI: 0.02–0.7)) (Table S2).

The clinical, biologic characteristics of two representative cases with different outcomes and SUV_max_ values are described in the Figure S1.

## 3. Discussion

PET/CT is widely used in the clinical management of CLL patients with disease progression and clinical suspicion of RS. This study was aimed at evaluating whether the SUV_max_ value could also have a prognostic impact on the outcome of patients with biopsy-proven CLL. The strength of this study was based on the homogeneity of the clinical characteristics of patients, which all showed progressive disease and performed PET/CT and the excisional biopsy of the involved lymph node or of another involved tissue before frontline therapy. The weaknesses of this study are the retrospective design, the relatively small sample size of patients, and the lack of a centralized review of biopsies and the PET/CT scans.

Clinical and histopathological signs suggesting a pronounced cell proliferation in lymph-nodes, such as large lymph-nodes, increased levels of lactate dehydrogenase (LDH), a high rate of Ki67 positive cells, and deletion 11q, were significantly more frequent in the presence of SUV_max_ values ≥ 5. The majority of patients achieved a response to front line therapy and the 18F-FDG uptake did not identify cases with a different response rate. Interestingly, in multivariate analysis, the presence of large and confluent PCs emerged as the only factor with a negative effect on both, PFS and OS. Deletion 11q, a genetic aberration frequently observed in cases with extensive lymphadenopathy [19], also revealed a significant and independent impact on PFS. SUV_max_ values ≥ 5 were associated with a significantly adverse impact on OS. Median survival was not reached in the patients with SUV_max_ values < 5, while it was significantly lower, 56 months, in those with SUV_max_ value ≥ 5. A close relationship between a high 18F-FDG uptake and inferior survival has been previously reported in other studies [6,8,9].

As these studies included treatment naïve as well as previously treated patients, and were mainly focused on the discriminatory power of PET/CT in identifying cases with RS, our data are not easily comparable [5,6,7,8,9,20,21]. In our study, we only considered patients with a biopsy-proven CLL performed before frontline therapy and excluded patients with RS. Given these characteristics, it is not surprising that in our study no patients showed SUV_max_ values ≥ 10. Nevertheless, our data, focused on patients with a less advanced disease, confirm the adverse impact of higher 18F-FDG uptake on survival. In a study by Mato et al. that included venetoclax-treated patients, increased SUV_max_ values were also associated with a trend to a significantly inferior survival [20]. As targeted therapy is progressively replacing chemotherapy, this question deserves to be further investigated in patients treated with B-cell receptor (BCR) and BCL2 antagonists. It might be possible that the 18F-FDG uptake loses its prognostic significance on survival when it will be tested in patients treated with BCR inhibitors that have a high efficacy on the lymph node disease.

Overall, our results suggest a close relationship between a high 18F-FDG uptake, histologic features of increased cell proliferation in lymph nodes, and survival probability. In a study by Ginè et al., patients whose biopsy displayed expanded PCs showed a low survival and were defined as having an ‘accelerated’ CLL [18]. Similarly, in a study by Falchi et al., patients with large and confluent PCs and high rates of Ki67 positive cells were classified as having a “histologically aggressive CLL” (HAC) [6]. Patients with a HAC revealed higher median SUV_max_ values and inferior survival than those without HAC features [6,9]. It is noteworthy that in our study, more than a third of the patients could be considered as patients with HAC at the time of the first treatment. An intriguing finding of the present study was the significantly higher rate of patients displaying SUV_max_ values ≥ 5 at baseline who developed a diffuse large B-cell lymphoma. Moreover, the majority of patients who developed RS also showed biopsies characterized by large and confluent PCs and high rates of Ki67 positive cells. This observation suggests that a pronounced cell proliferation in the lymph-node compartment could represent a risk factor for the expansion of an aggressive cell clone leading to the emergence of RS. This hypothesis has been supported by the high rate of genetic abnormalities that have been described in PCs [17,22].

Taken together, PET/CT is a useful exam providing an immediate representation of both the extent of the disease burden and the proliferating activity in the lymph node compartment, which is the preferential site of activation, and the proliferation of CLL cells [23]. Based on these properties, PET/CT is useful, not only in identifying patients with RS, but also CLL patients displaying a high rate of proliferating cells in the lymph node compartment and inferior survival.

## 4. Methods

### 4.1. Patients Population

Between January 2009 and January 2019, clinical, biologic, and radiologic data of CLL patients who performed PET/CT followed by the biopsy involved lymph nodes or different tissues were collected and retrospectively analyzed.

Inclusion criteria included: (1) the diagnosis of B-cell CLL, according to the international workshop on CLL (iwCLL) criteria [19]; (2) no prior treatment; (3) clinical and laboratory signs suggesting progressive disease requiring frontline therapy (bulky nodes, a rapid increase in the lymph-nodes size, extra-nodal involvement, B symptoms, increased LDH, progressive cytopenia) according to the iwCLL criteria [24]; (4) PET/CT followed by a biopsy of the involved lymph node or other tissue with the highest SUV_max_; (5) histologic diagnosis of CLL/small lymphocytic lymphoma; (6) signed informed consent from the patients permitting the collection of clinical data from medical records. Exclusion criteria included: (1) histologic transformation from CLL to an aggressive lymphoma (Richter transformation); (2) histologic diagnosis of second malignancy.

Response to treatment was assessed according to the iwCLL criteria [24]. The highest SUV_max_ value measured by PET provided the optimal site to perform the biopsy. In patients who showed the highest SUV_max_ projected on abdominal nodes, the largest and most easily reachable lymph node was biopsied. Forty patients with a biopsy showing a CLL/small lymphocytic lymphoma diagnosis were included in this study, while five patients, three with a RS, two with a second malignancy, were excluded (Figure S2).

The following data were analyzed: demographics (sex, age), disease stage, lymph-nodes size, complete blood count with differential, beta2-microglobulin, LDH, CD38 expression, IGHV mutation status, FISH cytogenetic profile (del 13q, del 11q, del 17p, +12), *TP*53 mutations. SUV_max_ values and histologic characteristics of the biopsied tissue were also considered. Other analyzed parameters included the type of treatment, response to treatment, disease progression, death, and causes of death.

### 4.2. PET/CT Imaging

PET/CT was performed by qualified radionuclide radiologists of nuclear medicine departments. Briefly, after at least eight-hour fasting, the patients were administered 4.0 ± 0.5 MBq/kg 18F-FDG and underwent the PET/CT scan (Discovery 710, General Electric, Milwakee, WI, USA) 60 ± 10 min later. Three-dimensional emission scans of 2.5 min per bed position were acquired from vertex to pelvis. Blood glucose levels were measured in all patients and 140 mg/dl was considered the upper limit for the 18F-FDG PET scan. Unenhanced low-dose CT (80–120 mAs using dose modulation, 80 kV, pitch of 0.938, and slice thickness of 3.75 mm) for segmented attenuation correction was carried out in each patient. Immediately after, the PET scans were acquired covering the same field of view as the CT. Images (256 × 256 matrix) were obtained with a 3D iterative ordered-subset expectation maximization (OSEM) and time of flight (TOF) technologies (GE VUE Point FX, GE Healthcare, Chicago, IL, USA). The algorithms were applied to the ratio sinograms using attenuation-weighted iterative reconstruction (three iterations, 18 subsets) and all the reconstructions always included a *z* axis filter. Ten millimeter-long circular region of interest (ROI) including 58 pixels was used to extract the standardized uptake value based on the body weight (SUVbw) values in a region with increased 18F-FDG uptake (Figure S3). A pathologic lesion with increased 18FDG uptake was defined as a pathologic area shown by TC with an increased uptake that appeared focal, and more intense than that of the adjacent background tissues.

The SUV_max_ values of involved lymph nodes or other involved tissues were recorded. Based on the results of our prior study [8], the patients’ characteristics and outcomes were analyzed according to a SUVmax cut-off of five or higher.

### 4.3. Histologic Analysis

An excisional lymph node biopsy was performed in all cases. The site with the highest SUV was considered to drive the biopsy to the most metabolically active area within a lymph node or to extra nodal tissue. Experienced pathologists of local academic institutions evaluated the histologic specimens. All the cases diagnosed as CLL/small lymphocytic lymphoma (SLL) were included in this study. The PCs were defined as described previously [18]. PCs with the major diameter exceeding the ax20 microscopic field were considered as large. Ki-67 index was evaluated by immunostaining (Dako, Glostrup, Denmark) in 8–10 PCs, as described previously [18]. A Zeiss Axiophot microscope (Carl Zeiss, Sheung kehen, Germany) was used.

### 4.4. Statistical Analysis

Patients’ characteristics were reported using the median for continuous variables and frequency for categorical variables. A chi-square test was used to assess the association between the categorical variables and the patients’ characteristics. Overall survival (OS) was calculated from the time of biopsy until death or last follow-up, progression-free survival (PFS) was calculated from the time of biopsy until disease progression, death, or last follow-up. The Kaplan–Meier method was considered to estimate the survival probabilities and differences between the survival curves assessed using the log-rank test. Univariate and multivariate Cox regression analyses were performed to evaluate the association between the patients’ characteristics and survival.

Statistical significance was considered for *p* values ≤ 0.05. All the calculations were made using a standard statistical package (SPSS for Windows Version 25.0; Chicago, IL, USA).

### 4.5. Ethics

This retrospective, observational study was carried out according to the Guidelines for Good Clinical Practice and was approved by the Ethics Committee (Rif.3342/11.09/2014). Patients were asked to provide informed consent.

## 5. Conclusions

Our results show that higher SUV_max_ values identify CLL patients with a pronounced rate of proliferating cells in the lymph-node compartment, inferior survival, and an increased risk of developing RS. The prognostic significance of PET/CT deserves to be further investigated in trials, including the CLL patients treated with novel agents.

## Figures and Tables

**Table 1:** Clinical and biologic characteristics of the patients with biopsy-proven chronic lymphocytic leukemia (CLL) according to the SUV_max_ values.

Tested Variables	All Patients *n* (%)	SUV_max_ ≥ 5 *n* (%)	SUV_max_ < 5 *n* (%)	*p* Value
	40 (100)	13 (33)	27 (67)	-
Age				
≥65 years	14 (35)	6 (46)	8 (30)	0.30
<65 years	26 (65)	7 (54)	19 (70)	
Sex				
Male	31 (78)	12 (92)	19 (70)	0.12
Female	9 (22)	1(8)	8 (30)	
Binet stage				
A+B	34 (85)	11 (85)	23 (85)	0.96
C	6 (15)	4 (15)	2 (15)	
B symptoms				
Present	11 (28)	3 (23)	8 (30)	0.66
Absent	29 (72)	10 (77)	19 (70)	
LDH				
Normal	13 (33)	6 (50)	20 (74)	0.14
Increased	26 (67)	6 (50)	7 (26)	
Beta 2 microglobulin				
≥3.5 mg/L	21 (58)	4 (40)	11 (42)	0.9
<3.5 mg/L	15 (42)	6 (60)	15 (58)	
Lymph nodes diameter				
≥5 cm	17 (46)	8 (62)	9 (38)	0.16
<5 cm	20 (54)	5 (38)	15 (62)	
Large and confluent PCs				
Present	9 (24)	4 (36)	5 (19)	0.26
Absent	28 (76)	7 (64)	21 (81)	
Ki67%				
≥30%	15 (40)	8 (67)	7 (27)	0.02
<30%	23 (60)	4 (33)	19 (73)	
CD38 positive				
Present	18 (47)	7 (58)	11 (44)	0.41
Absent	19 (51)	5 (42)	14 (56)	
Unmutated IGHV				
Present	21 (54)	9 (75)	12 (44)	0.07
Absent	18 (46)	3 (25)	15 (56)	
FISH ^b^				
Del(11q)				
Present	6 (19)	5 (45)	1 (5)	0.006
Absent	25 (81)	6 (55)	19 (95)	
Trisomy 12				
Present	8 (26)	4 (36)	4 (20)	0.32
Absent	23 (74)	7 (64)	16 (80)	
Del(13q)				
Present	5 (16)	1 (9)	4 (20)	0.43
Absent	26 (84)	10 (91)	16 (80)	
Del(17p) and/or mutated *TP*53 ^c^				
Present	7 (19)	3 (27)	4 (15)	0.39
Absent	30 (81)	8 (72)	22 (85)	

Abbreviations: BR, bendamustine, rituximab; Del, deletion; F, female; FCR, fludarabine, cyclophosphamide, rituximab; LDH, lactate dehydrogenase; PCs, proliferation centers; SUV, Standardized uptake value; WBC, white blood cell. Extra-nodal involvement in 3 patients: skin two cases, gastric one case. ^b^ FISH investigated in 31 patients, *TP*53 mutation investigated in 37. ^c^ Deletion 17p detected in 3/31 patients. *TP*53 mutation detected in 6/37 patients (2 patients with deletion 17p also had TP53 mutation).

**Table 2:** PFS and OS according to the baseline characteristics of the patients: univariate analysis.

Tested Variables	No. Patients *n* (%)	PFS				OS			
		3 Year %	Median Months	*p* Value	HR (95% CI)	3 Year %	Median Months	*p* Value	HR (95% CI)
	40 (100)	75	48	-	-	94	-	-	-
SUV_max_									
≥5	13 (33)	55	37	0.48	0.72 (0.2–1,7)	83	56	0.002	0.12 (0.3–0.5)
<5	27 (67)	79	49			100	-		
Age									
≥65 years	14 (35)	62	47	0.78	1.13 (0.4–2.7)	84	126	0.33	1.81 (0.5–6)
<65 years	26 (65)	75	48			100	-		
Sex									
Male	31 (78)	68	47	0.44	0.65 (0.2–1.9)	100	-	0.21	0.02 (0–8.6)
Female	9 (22)	85	53			93	-		
B symptoms									
Present	11 (28)	72	47	0.57	1.28 (0.5–3)	100	126	0.37	0.50 (0.1–2.3)
Absent	29 (72)	71	48			92	-		
Binet stage									
A+B	34 (85)	71	47	0.45	0.57 (0.1–2.4)	93	-	0.65	0.62 (0.8–4.9)
C	6 (15)	75	49			100	-		
LDH									
Normal	13 (33)	70	62	0.31	1.56 (0.6–3.6)	91	-	0.75	0.80 (0.2–3.1)
Increased	26 (67)	74	42			90	-		
Beta 2 microglobulin									
≥3.5 mg/L	21(58)	77	53	0.31	6.35 (0.2–1.5)	92	-	0.94	0.95 (0.2–3.5)
<3.5 mg/L	15 (42)	64	42			95	-		
Lymph nodes diameter									
≥5 cm	17 (46)	67	44	0.78	1.1 (0.4–2.6)	94	-	0.35	1.7 (0.5–5.8)
<5 cm	20 (54)	73	47			93	-		
Large and confluent PCs									
Present	9 (24)	30	35	0.003	4.50 (1.6–12.1)	85	59	0.02	5.17 (1.2–21.1)
Absent	28 (76)	85	62			96	0		
Ki67%									
≥30%	15 (40)	58	40	0.04	2.41 (1–5.6)	93	126	0.009	8.1 (1.7–38.6)
<30%	23 (60)	85	53			100	-		
CD38+									
Present	18 (47)	65	49	0.93	0.96 (0.3–2.4)	92	126	0.20	2.19 (0.6–7.2)
Absent	19 (51)	73	47			89	-		
Unmutated IGHV									
Present	21 (54)	59	40	0.07	0.43 (0.2–1)	95	-	0.04	0.20 (0.04–0.9)
Absent	18 (46)	86	53			100	-		
Del(17p) and or mutated *TP*53									
Present	7 (19)	57	47	0.70	1.21 (0.4–3.3)	83	126	0.71	1.31 (0.3–5.6)
Absent	30 (81)	76	48			96	-		
Del(11q)									
Present	6 (19)	75	32	0.01	4.21 (1.3–13)	83	40	0.01	5.49 (1.4–20.8)
Absent	25 (81)	40	48			100	-		
Trisomy 12									
Present	8 (26)	85	49	0.43	0.64 (0–1.9)	100	-	0.93	0.40 (0.05–3.2)
Absent	23 (74)	64	47			95	126		
Del(13q)									
Present	4(14)	50	35	0.20	2.04 (0.6–6.1)	100	-	0.60	0.57 (0.07–4.5)
Absent	27 (86)	72	47			95	126		

Abbreviations: BR, bendamustine, rituximab; Del, deletion; FCR, fludarabine, cyclophosphamide, rituximab; HR, hazard ratio; LDH, lactate dehydrogenase; PCs, proliferation centers; OS, overall survival; PFS, progression-free survival; SUV, standardized uptake value.

## Supplementary Materials

The following are available online at https://www.mdpi.com/2072-6694/12/7/1773/s1, Figure S1: Two representative cases with different SUV_max_ values, Figure S2: Consort diagram-trial profile, Figure S3: Example of ROI positioning, Table S1: Response to treatment, CLL progression, Richter syndrome according to the SUV_max_ values, Table S2: Multivariable Cox regression model for PFS and OS.
