# Supplementary material for: Prognostic Significance of PET/CT in Patients with Chronic Lymphocytic Leukemia (CLL) Treated with Frontline Chemoimmunotherapy
Source: Cancers (Basel). 2020 Jul 3;12(7):1773. doi: 10.3390/cancers12071773 (PMC7408608; doi:10.3390/cancers12071773)
Supplement: Supplementary file 1 [file cancers-12-01773-s001.pdf]

cancers

Article

Prognostic Significance of PET/CT in Patients with Chronic Lymphocytic Leukemia (CLL) Treated with Frontline Chemoimmunotherapy

Marika Porrazzo, Emanuele Nicolai, Mara Riminucci, Candida Vitale, Marta Coscia, Lorenzo De Paoli, Angela Rago, Giulia Buscicchio, Giacomo Maestrini, Silvio Ligia, Alessio Di Prima, Alessandro Corsi, Roberto Caronna, Gianluca Gaidano and Francesca Romana Mauro

Supplementary Materials:

CASE #1 - Female , 75 years.<br><br>Biologic profile: IGHV, mutated; FISH, no aberrations; *TP53*, no mutation.<br><br>^{18}F-FDG uptake: SUV_{max} value=3.4 (submandibular enlarged lymph nodes)<br><br>Histology: CLL/SLL; no large and confluent PCs; Ki67+ cells 15%.<br><br>Therapy: FCR. Response: CR.<br><br>Alive, stable disease (+54 months).	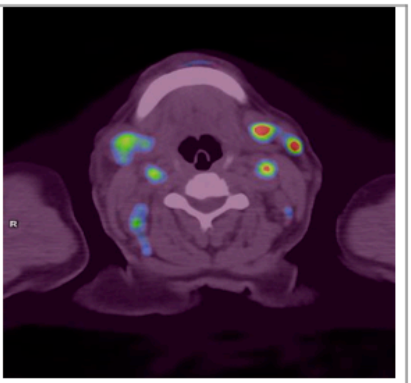
CASE #2 - Male , 62 years.<br><br>Biologic profile: IGHV, unmutated; FISH, del(11q); complex karyotype, *TP53*, no mutation.<br><br>^{18}F-FDG uptake: SUV_{max} value=7.8 (submandibular enlarged lymph nodes)<br><br>Histology: CLL/SLL; large and confluent PCs; Ki67+ cells 30%.<br><br>Therapy :FCR. Response: PR.<br><br>Died due to Richter transformation (= 32 months).	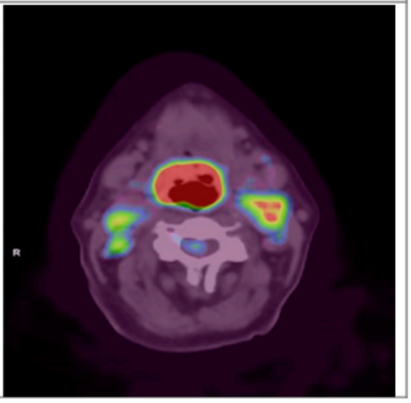

Abbreviations: CLL, chronic lymphocytic leukemia; CR, complete response; FCR, fludarabine, cyclophosphamide,rituximab; ^{18}F-FDG, ^{18}F fluorodeoxyglucose ; FISH, fluorescence in situ hybridisation; PCs, proliferation centers; PR, partial response; SUV, Standardized Uptake Value.

Figure S1. Two representative cases with different SUV_{max} values.

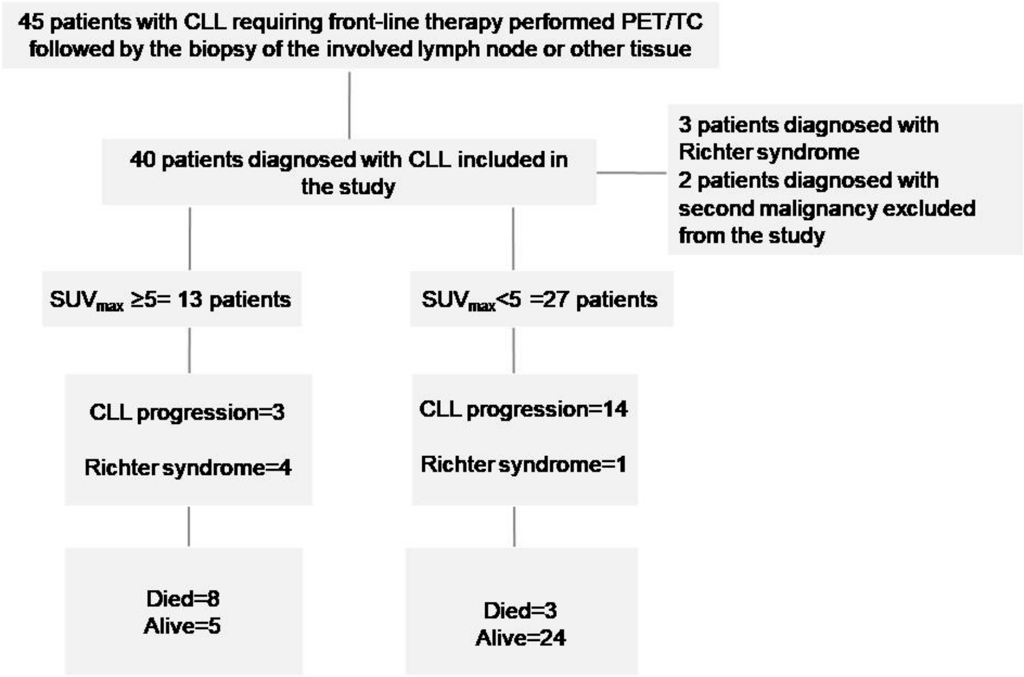

Figure S2. Consort diagram-trial profile.

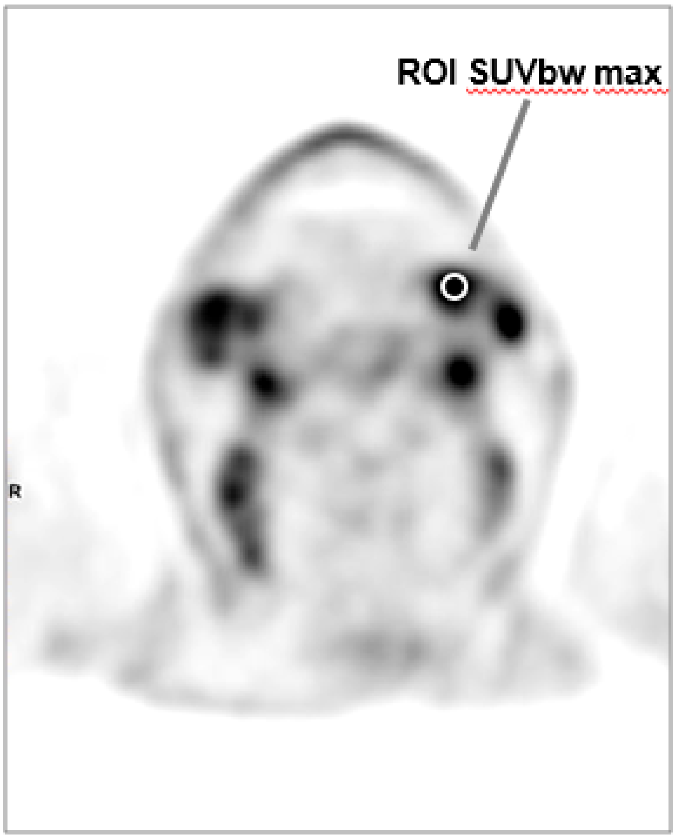

Figure S3. Example of ROI positioning.

Table S1. Response to treatment, CLL progression, Richter syndrome according to the SUV_{max}values.

	**All Patients**	**SUV$_{max}$ ≥ 5**	**SUV$_{max}$ < 5**	***p* Value**
No patients (%)	40 (100)	13 (33)	27 (67)	-
Response to treament				
Yes	36 (90)	11 (85)	25 (93)	0.43
No	4 (10)	2 (15)	2 (7)	
CLL progression[(a)]				
Yes	17 (49)	3 (33)	14 (54)	0.28
No	18 (51)	6 (67)	12 (46)	
Richter syndrome				
Yes	5 (13)	4(31)	1 (4)	0.015
No	35 (87)	9 (69)	26 (96)	

Table S2. Multivariable Cox regression model for PFS and OS.

**PFS**			**OS**		
**Tested Variables**	**HR (95%CI)**	***p*-Value**	**Tested Variables**	**HR (95%CI)**	***p*-Value**
Large and confluent PCs present vs. absent	0.17 (0.03-0.94)	0.04	Large and confluent PCs present vs. absent	0.14 (0.02–0.7)	0.02
Del(11q) present vs. absent	0.28 (0.08-0.99)	0.05	Del(11q) present vs. absent	2.54 (0.22–29.19)	0.34
Ki67 ≥30% vs. Ki67 <30%	1.45 (0.31 -6.8)	0.63	Ki67 ≥30% vs. <30%	1.50 (0.08–28.53)	0.94
			IGHV unmutated vs. mutated	0.53 (0.08–3.66)	0.36
			SUV$_{max}$ ≥ 5 vs. x < 5	6.48 (1.42–29.58)	0.02

© 2020 by the authors. Submitted for possible open access publication under the terms and conditions of the Creative Commons Attribution (CC BY) license (http://creativecommons.org/licenses/by/4.0/).
